# Supplementary material for: The Protective Effect of Zebularine, an Inhibitor of DNA Methyltransferase, on Renal Tubulointerstitial Inflammation and Fibrosis
Source: Int J Mol Sci. 2022 Nov 14;23(22):14045. doi: 10.3390/ijms232214045 (PMC9697081; doi:10.3390/ijms232214045)
Supplement: Supplementary file 1 [file ijms-23-14045-s001.zip › Supplementary Figure S1.pdf]

Supplementary Figure S1. The effect of zebularine on the expression of IL-10 and IL-11 mRNAs in obstructed kidneys

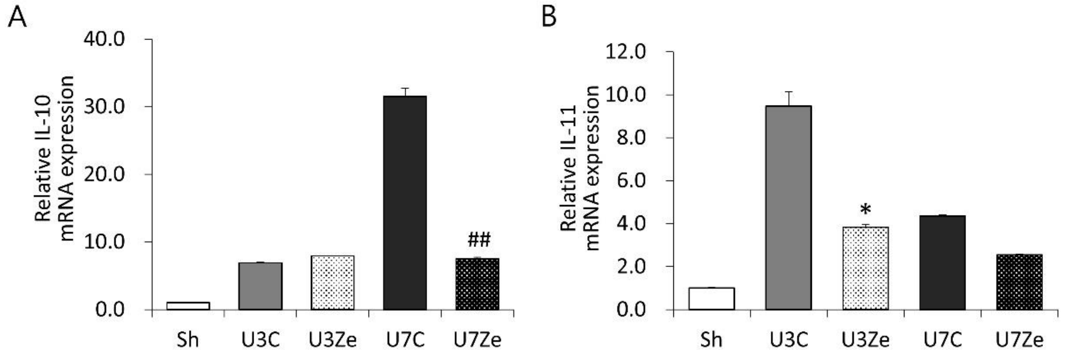

The mRNA expression of *IL-10* (A) and *IL-11* (B) was measured by qRT-PCR as described in Materials and Methods using GAPDH mRNA as the internal reference gene. QRT-PCR was performed independently three times, each reaction of which was performed in duplicate. The results were presented as mean ± standard error of the mean (SEM).
