# Supplementary material for: The Protective Effect of Zebularine, an Inhibitor of DNA Methyltransferase, on Renal Tubulointerstitial Inflammation and Fibrosis
Source: Int J Mol Sci. 2022 Nov 14;23(22):14045. doi: 10.3390/ijms232214045 (PMC9697081; doi:10.3390/ijms232214045)
Supplement: Supplementary file 1 [file ijms-23-14045-s001.zip › Supplementary Figure S3.pdf]

Supplementary Figure S3. The images of negative IHC

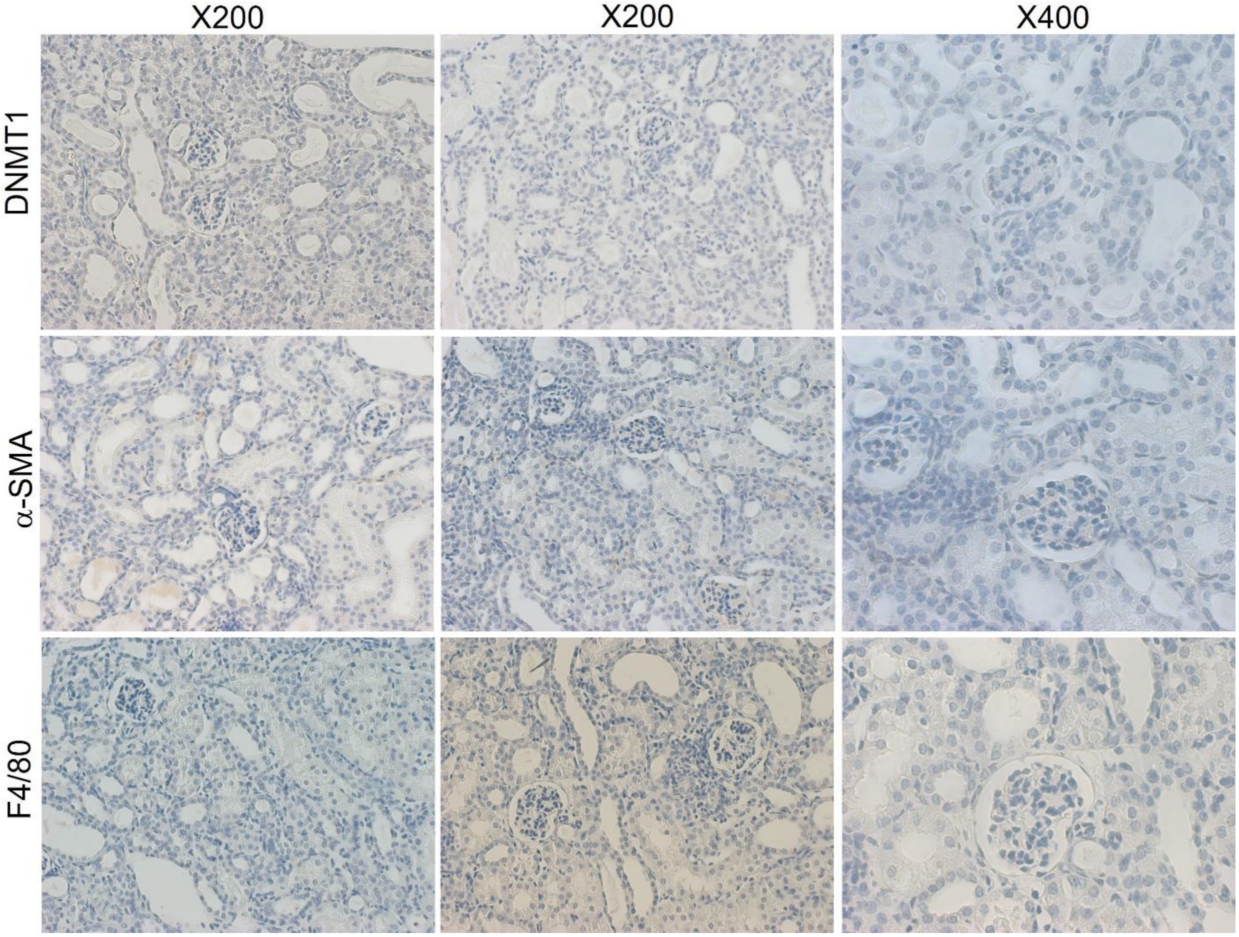

To include the negative control for Figure 1A (Dnmt1), we conducted IHC against U7C which showed the highest level of Dnmt1 expression, by skipping anti-Dnmt1 antibody incubation. Briefly, a section of U3C tissue were incubated in primary antibody incubating solution without anti-DNMT1 antibody. After washing in PBS, the section was incubated for 60 min with peroxidase-conjugated anti-rabbit IgG (Jackson ImmunoResearch Laboratories, West Grove, PA, USA) as a secondary antibody and then reacted with a mixture of 3,3′-diaminobenzidine (0.05%)-containing H_2O_2 (0.01%) for the colour reaction. After counterstaining with haematoxylin, the section was photographed.

To include the negative control for Figure 3A (α-SMA), we conducted IHC against U7C which showed the maximum level of a-SMA protein, by skipping anti-α-SMA antibody incubation. Briefly, a section of U7C tissue were incubated in primary antibody incubating solution without anti-α-SMA antibody. After washing in PBS, the section was incubated for 60 min with peroxidase-conjugated anti-mouse IgG (Jackson ImmunoResearch Laboratories, West Grove, PA, USA) as a secondary antibody and then reacted with a mixture of 3,3′-diaminobenzidine (0.05%)-containing H_2O_2 (0.01%) for the colour reaction. After counterstaining with haematoxylin, the section was photographed.

To include the negative control for Figure 4A (F4/80), we conducted IHC against U7C which showed the highest positivity, by skipping anti-F4/80 antibody incubation. Briefly, a section of U7C were incubated in

primary antibody incubating solution without anti-F4/80 antibody. After washing in PBS, the section was incubated for 60 min with peroxidase-conjugated anti-rat IgG (Jackson ImmunoResearch Laboratories, West Grove, PA, USA) as a secondary antibody and then reacted with a mixture of 3,3′-diaminobenzidine (0.05%)-containing H_2O_2 (0.01%) for the colour reaction. After counterstaining with haematoxylin, the section was photographed
