# Supplementary material for: The Protective Effect of Zebularine, an Inhibitor of DNA Methyltransferase, on Renal Tubulointerstitial Inflammation and Fibrosis
Source: Int J Mol Sci. 2022 Nov 14;23(22):14045. doi: 10.3390/ijms232214045 (PMC9697081; doi:10.3390/ijms232214045)
Supplement: Supplementary file 1 [file ijms-23-14045-s001.zip › Supplementary Table S1.pdf]

Supplementary Table S1. Sequences of primers for qRT-PCR against indicated genes

Gene	Forward (5′→3′)	Reverse (5′→3′)
TGF-β1	GAC CGC AAC AAC GCC ATC TA	GGC GTA TCA GTG GGG GTC AG
α-SMA	GGA GAA GCC CAG CCA GTC GC	AGC CGG CCT TAC AGA GCC CA
Vimentin	CGG AAA GTG GAA TCC TTG CA	CAC ATC GAT CTG GAC ATG CTG T
Fibronectin	GAA GTC GCA AGG AAA CAA GC	GTT GTA GGT GAA CGG GAG GA
MMP-2	GCC TCA TAC ACA GCG TCA ATC TT	CGG TTT ATT TGG CGG ACA GT
MMP-9	CCT GGA ACT CAC ACG ACA TCT TC	TGG AAA CTC ACA CGC CAG AA
FSP-1	GGA GCT GCC TAG CTT CCT G	GCT GTC CAA GTT GCT CAT CA
N-cadherin	ATG GCC TTT CAA ACA CAG CCA CAG	ACA ATG ACG TCC ACC CTG TTC TCA
E-cadherin	ATC CTC GCC CTG CTG CTT	ACC ACC GTT CTC CTC CGT A
Snail1	ACA GCTGCT TCG AGC CAT AGA ACT	TGT ACC TCA AAG AAG GTG GCC TGA
IL-1β	CAA CCA ACA AGT GAT ATT CTC CAT G	ATC CAC ACT CTC CAG CTG CA
IL-6	TGT ATG AAC AAC GAT GAT GCA	GGT ACT CCA GAA GAC CAG AGG AAA T
IL-17	CAG GGA GAG CTT CAT CTG TGT	GCT GAG CTT TGA GGG ATG AT
TNF-α	CCC AGA CCC TCA CAC TCA GAT C	CCT CCA CTT GGT GGT TTG CT
IL-10	CAG AGC CAC ATG CTC CTA GA	TGT CCA GCT GGT CCT TTG TT
IL-11	AAT TCC CAG CTG ACG GAG ATC ACA	TCT ACT CGA AGC CTT GTC AGC ACA
Dnmt1	CAG AGA CTC CCG AGG ACA GA	TTT ACG TGT CGT TTT TCG TCT C
Dnmt3b	CCC TCC CCC ATC CAT AGT	TCT GCT GTC TCC CTT CAT TGT
