# Supplementary figures and images for: The Protective Effect of Zebularine, an Inhibitor of DNA Methyltransferase, on Renal Tubulointerstitial Inflammation and Fibrosis
Source: Int J Mol Sci. 2022 Nov 14;23(22):14045. doi: 10.3390/ijms232214045 (PMC9697081; doi:10.3390/ijms232214045)

**Supplementary Figure S2.** The action mechanism of zebularine on the UUO-induced fibrosis

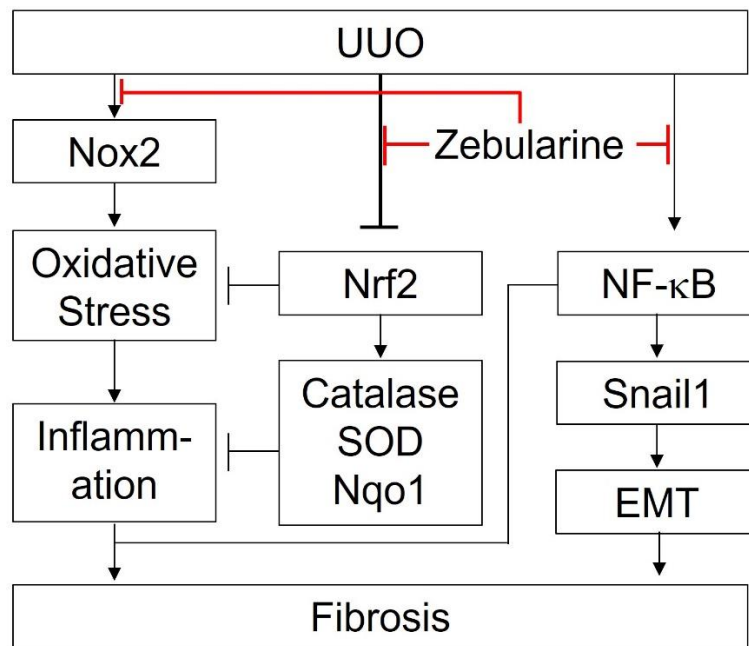

Supplement: Supplementary file 1 [file ijms-23-14045-s001.zip › Supplementary Figure S2.pdf]
